# Biotechnological potentials of surfactants in coal utilization: a review

**DOI:** 10.1007/s11356-024-34892-5

**Published:** 2024-09-07

**Authors:** Nuraly Akimbekov, Ilya Digel, Azhar Zhubanova, Kuanysh T. Tastambek, Atakan Tepecik, Dinara Sherelkhan

**Affiliations:** 1 Scientific-Practical Center, West Kazakhstan Marat Ospanov Medical University, Maresyev str. 68, Aktobe, 030019 Kazakhstan; 2 Sustainability of Ecology and Bioresources, Al-Farabi Kazakh National University, Al-Farabi ave. 71, Almaty, 050040 Kazakhstan; 3 Ecology Research Institute, Khoja Akhmet Yassawi International Kazakh-Turkish University, Sattarhanov str. 29, Turkistan, 161200 Kazakhstan; 4 Institute for Bioengineering, Aachen University of Applied Sciences, Heinrich-Mussmann-Straße 1, Jülich, 52428 Germany

**Keywords:** Coal, Surfactants, Microorganisms, Biosolubilization, Biobeneficiation, Methanogenesis, Coal dust suppression

## Abstract

The quest for scientifically advanced and sustainable solutions is driven by growing environmental and economic issues associated with coal mining, processing, and utilization. Consequently, within the coal industry, there is a growing recognition of the potential of microbial applications in fostering innovative technologies. Microbial-based coal solubilization, coal beneficiation, and coal dust suppression are green alternatives to traditional thermochemical and leaching technologies and better meet the need for ecologically sound and economically viable choices. *Surfactant-mediated approaches* have emerged as powerful tools for modeling, simulation, and optimization of coal-microbial systems and continue to gain prominence in clean coal fuel production, particularly in microbiological co-processing, conversion, and beneficiation. Surfactants (surface-active agents) are amphiphilic compounds that can reduce surface tension and enhance the solubility of hydrophobic molecules. A wide range of surfactant properties can be achieved by either *directly* influencing microbial growth factors, stimulants, and substrates or *indirectly* serving as frothers, collectors, and modifiers in the processing and utilization of coal. This review highlights the significant biotechnological potential of surfactants by providing a thorough overview of their involvement in coal biodegradation, bioprocessing, and biobeneficiation, acknowledging their importance as crucial steps in coal consumption.

## Introduction

*Coal*, playing a dual role as a primary energy source and key industrial raw material, has massively contributed to the economic growth of numerous nations worldwide. Recently, the scale of mining and coal processing has expanded considerably due to rapid growth and continuous advancements in automation within the coal industry. Nevertheless, there is increasing worldwide apprehension over the impact of coal consumption on environmental degradation, global warming, and climate change. The importance of research on *clean coal technologies* has been emphasized within the broader sustainability framework of energy production and utilization, with a focus on safety and environmental preservation (Akimbekov et al. 2023; Finkelman et al. 2021; Osborne et al. 2023). Microbiological co-processing, conversion, and beneficiation are examples of microbial-based clean coal technologies necessary for achieving considerably greener mitigation solutions and addressing the environmental challenges that arise at various stages of coal consumption (Mishra et al. 2015; B. Wang et al. 2019a, b, c; X. Wang et al. 2023a, b).

In recent years, surfactants or surface-active agents (primarily those derived from microorganisms) have been used in a variety of coal-utilization applications. Their utilization stems from their potential to make conventional energy generation more environmentally friendly, efficient, and sustainable. Surfactants exhibit impressive functional diversity, acting as emulsifiers (forming of emulsion), stabilizers, promoters, and collectors (absorbing on the mineral surface) in coal technologies (Chang et al. 2020; Guin and Singh 2023). Biosurfactants produced by various microorganisms offer great promise in the coal industry due to reduced adverse environmental impacts and improved utilization safety because of their biodegradability, nontoxicity, stability, and specific activity (Bhadra et al. 2023; Maddela et al. 2023; Oyetunji et al. 2023).

Biosurfactants are amphiphilic molecules, possessing both hydrophilic and hydrophobic moieties. The hydrophilic portion often consists of carbohydrates, amino acids, peptide anions/cations, or phosphate groups. The hydrophobic part typically comprises an elongated tail of saturated or unsaturated, linear or branched fatty acids (Banat et al. 2010; Carolin C et al., 2023). Surface-active agents reduce surface tension at liquid–liquid or liquid–solid interfaces (Pacwa-Płociniczak et al. 2011). Biosurfactants, a diverse array of secondary metabolites, are crucial to microbial survival due to the enhancement of the bioavailability of hydrophobic substrates, mediation of microbe-host interactions, and participation in quorum sensing (the ability to regulate gene expression according to population density) mechanisms. In addition, they can function as antimicrobial, insecticidal, antibiofilm, and anti-adhesive agents (Inès and Dhouha 2015). Biosurfactants can be classified according to their ionic charges (anionic, cationic, non-ionic, and neutral), molecular weight (high and low molecular weight), and secretion type (intracellular, extracellular, and adherent to microbial cells) (Marchant and Banat 2012). In 1941, Bushnell and Hass were the pioneers in demonstrating the bacterial synthesis of biosurfactants. They achieved this by cultivating *Corynebacterium* and *Pseudomonas* strains in mineral media (Ilori et al. 2008). Sophorolipids are extracellular biosurfactants produced by certain *Starmerella* strains, which were first discovered in the early 1960s (Jiménez-Peñalver et al. 2019; H. Wang et al. 2019a, b, c). Rhamnolipids, glycolipid-type biosurfactants produced by *Pseudomonas aeruginosa*, are among the initial biosurfactants identified for their environmental significance (Edwards and Hayashi 1965). Since then, a multitude of research has been conducted to investigate the nature and function of biosurfactants in coal processing and utilization (Chong and Li 2017).

Synthetic surfactants, mainly anionic and nonionic, are widely used in coal bioprocessing. Nonionic surfactants have the potential to enhance coal stability and hydrophilicity, accelerate degradation, and promote microbial growth. However, their high cost and required high dosage pose practical problems (Jiang et al. 2013; Shi et al. 2023a). Conversely, anionic surfactants are cost-effective and require lower dosages, but may exhibit poor solubility and often adhere readily to coal surfaces (Polman et al. 1994; West and Harwell 1992). For instance, sodium dodecyl sulfate (SDS), an anionic surfactant, enhances the charge and hydrophilicity of coal surfaces, thus facilitating the degradation of coal by extracellular enzymes (Yin et al. 2011). Another anionic surfactant, sodium dodecyl benzene sulfonate (LAS), stimulates coal-bacteria interactions, which leads to shorter degradation times (Kang et al. 2021). In general, nonionic surfactants tend to stimulate the proliferation of bacteria, whereas anionic surfactants rather facilitate coal-bacteria interactions. Occasionally, a combination of the two surfactant types can be used in coal processing to enhance the benefits offered by each (Shi, Liu, Wu, et al., 2023a; Zhang et al. 2020).

The environmental effects of synthetic surfactants are of major concern because of their toxicity to living organisms and biological processes (Chen et al. 2018; Fei et al. 2020). For instance, the presence of surfactants in water can lead to the formation of stable foam on the surface, which reduces the amount of sunlight that reaches the seabed due to the turbid nature of foam, which impacts photosynthesis in plants (Effendi et al. 2017). Several studies have shown that their negative effects can be alleviated by using microorganisms that can degrade specific surfactants via bioremediation (Bubenheim et al. 1997). However, due to their molecular structure, synthetic surfactants exhibit partial biodegradability when released into the environment. As a result, they persist in natural systems for extended periods and can accumulate in sediments and soils (Pradhan and Bhattacharyya 2017). Biosurfactants, as mentioned earlier, differ from manufactured surfactants, consisting of naturally occurring compounds synthesized by microorganisms. This distinctive composition confers advantageous characteristics, such as enhanced biodegradability and reduced toxicity, while retaining surface attributes comparable to synthetic materials (Uchegbu et al. 2013).

Biosurfactants for coal applications possess numerous advantages that facilitate their implementation in coal industries from a macro perspective. Biosurfactants can be employed in a variety of forms, including biosurfactant crude extract, purified biosurfactant, and biosurfactant-producing microorganisms (Eras-Muñoz et al. 2022). Conversely, the expenses associated with biosurfactants are contingent upon the availability of substrates, the activity of microorganism-producers, and production constraints. Nevertheless, certain authors have reported on emerging technologies, such as nanotechnologies and novel purification techniques, which are critical steps in obtaining a suitable product quality (Dolman et al. 2017; Venkataraman et al. 2022). Their industrial approach is regarded as an open research field (Sahebnazar et al. 2018).

In this review, we attempted to analyze and provide an overview of the most recent and promising surfactant-mediated microbial technologies, as well as the main methodological concepts proposed for the solubilization, functional transformation, and beneficiation of coal. To our knowledge, this is the first review comprehensively addressing the role of surfactants in microbial-based technologies for producing clean coal fuels.

## Surfactant-mediated coal biosolubilization

Biosolubilization, an environmentally sustainable method for utilizing low-rank coal, has garnered significant scientific interest (N. Akimbekov et al. 2021a, b). Humic acid, produced as a byproduct of coal biosolubilization/bioconversion, can significantly enhance soil quality. This approach demonstrates both feasibility and environmental friendliness due to its moderate operating conditions, minimal energy requirements, and uncomplicated equipment (N. S. Akimbekov et al. 2021a, b).

Three primary approaches are currently being explored to enhance coal biosolubilization: strain selection, coal pretreatment, and supplementation with active ingredients such as surfactants and enzymes (Ghani et al. 2015; Yuan et al. 2006). Among these, the use of surfactants seems to be especially promising, offering unique advantages over coal pretreatment and strain selection, such as ease of use and high efficiency. By increasing the hydrophobicity of the cell surface, surfactants facilitate the adsorption of hydrocarbons by microorganisms (Bezza and Chirwa 2017). Furthermore, surfactants can alter the cell membrane permeability, aiding the absorption of coal molecules and the release of microbial enzymes. Additionally, by reducing the surface tension at the coal surface, surfactants can enhance its solubility (Shen et al. 2023; Yuan et al. 2006).

Most surfactants used today are produced by chemical synthesis from petroleum-based resources. However, these surfactants may have harmful impacts on the environment since they are ecotoxic and only partly biodegradable (Vaz et al. 2012). The rise in environmental consciousness has led to a greater need for bio-based surfactants due to their potential to decrease the prevalence of synthetic analogs and alleviate their associated toxicity. Biosurfactants possess numerous advantages in coal solubilization compared to their artificial counterparts due to their superior biodegradability, minimal toxicity, and production from renewable substrates (Kiran et al. 2010). However, the positive effects of surfactants on coal biosolubilization have not always been consistent in previous research. On the one hand, in the experiments by Yuan et al. and Polman et al., surfactants have been shown to favorably influence coal biosolubilization in coal-surfactant-microorganism/enzyme systems (Polman et al. 1995; Yuan et al. 2006). On the other hand, Breckenridge et al. and Polman et al. in another (earlier) study reported that utilizing only coal-surfactant systems without microorganisms/enzymes demonstrated no effects (Breckenridge and Polman 1994; Polman et al. 1994). Substantial levels of coal biosolubilization induced by surfactants can be probably primarily attributed to the synergistic interaction among coal, surfactants, and microorganisms/enzymes, rather than the interaction between coal and surfactants alone.

**Fig. 1:**
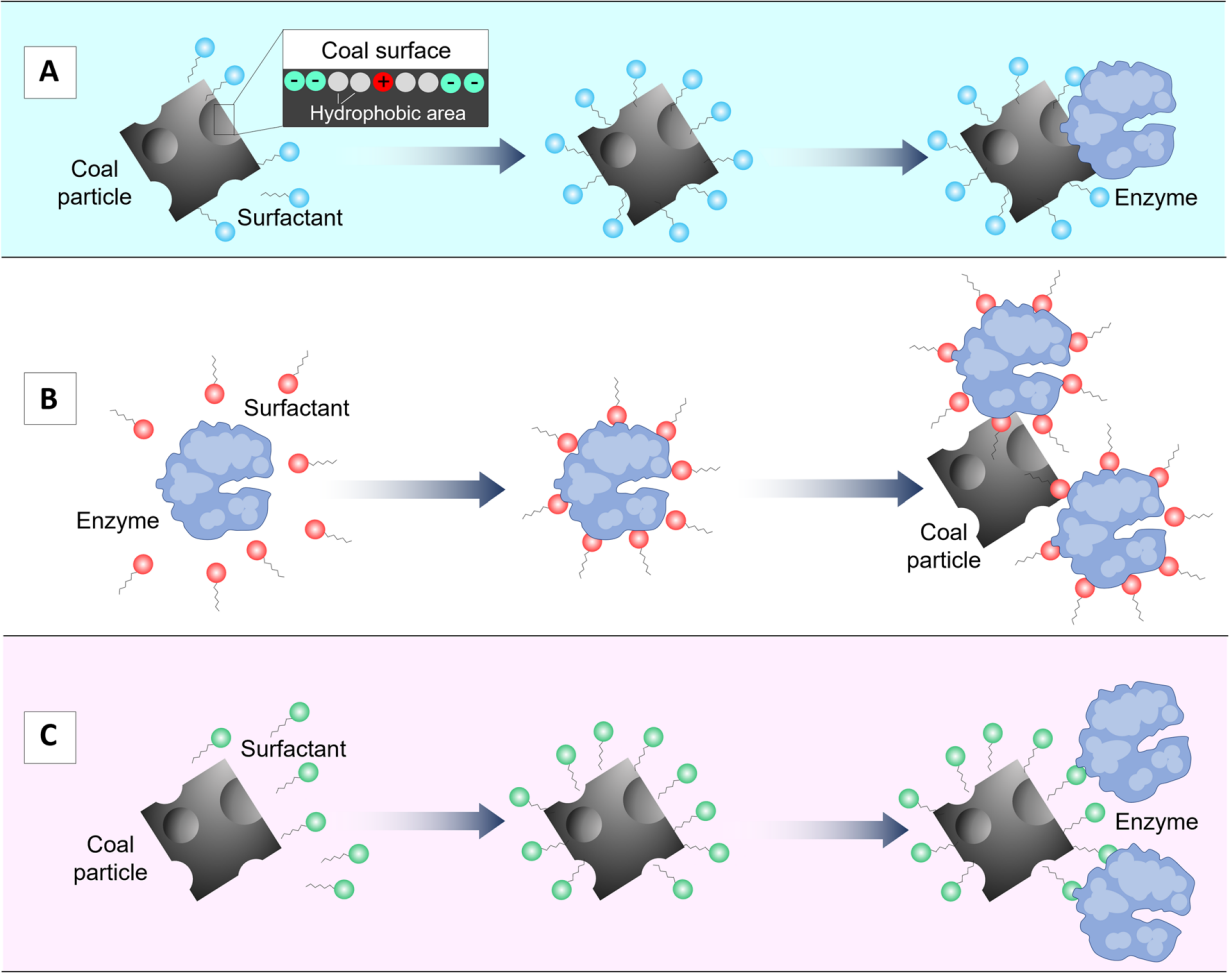
Three possible mechanisms of interactions between surfactants, enzymes, and coal particles during coal biosolubilization. **A** Surfactants emulsify the hydrophobic components of coal, facilitating subsequent breakdown by enzymes into smaller coal particles. **B** Formation of surfactant-enzyme complexes, which then adhere to the coal surface and enhance coal biosolubilization. **C** Surfactants initially adhere to the coal surface, followed by enzyme absorption, finally leading to coal solubilization via the hydrophilic portions of surfactants

Yin et al. proposed three principal interaction mechanisms (which do not exclude each other) between coal, surfactants, and enzymes (Yin et al. 2011). According to the first explanation (Fig. 1A), the surfactants first emulsify some hydrophobic components of coal. Subsequently, enzymes break down the emulsified tiny coal particles. In the second interaction model (Fig. 1B), enzyme-surfactant complexes first form in the liquid phase and then adhere to the coal surface, leading to coal biosolubilization. Finally, surfactants can first adhere to the coal surface (Fig. 1C) and solubilize it via the hydrophilic moieties; then enzymes are attached to the coal surface. Shi et al. demonstrated the combined action of an esterase enzyme and a rhamnolipid surfactant to promote coal biodegradation by *Pseudomonas japonica* (Shi, Liu, Zhao, et al., 2023b). According to their study, the adsorption of rhamnolipid by the cell surface increases the permeability of the cell membrane, allowing microorganisms to secrete more esterase, while rhamnolipids increase the electronegativity of coal by adsorbing onto its surface. Consequently, these processes degrade coal macromolecules into aromatic compounds, alcohols, ethers, and long-chain alkanes.

Numerous studies have demonstrated that biosurfactants can enhance the coal solubilization rate, which positions them as a promising tool for humic substance production and hydrocarbon bioremediation at polluted sites (Table 1).

**Table 1:** Microbially produced biosurfactants and their effects on coal solubilization

Microorganism	Source	Produced biosurfactant	Coal type	Reported effects	Prospects	Ref
*Pseudomonas stutzeri*	The formation water of an Indian coalbed	Rhamnolipids	Lignite, bituminous, and anthracite	With lignite, *P. stutzeri* produced more rhamnolipid than with bituminous or anthracite	*P. stutzeri* may be beneficial in in situ coal biotransformation into methane and the PAHs bioremediation	(Singh and Tripathi 2013)
*Candida bombicola*	Bumblebee honey	Sophorolipids	NS	The biosurfactant contributed marginally to the solubilization of coal	The potential engineering applications of biosurfactants was determined	(Breckenridge and Polman 1994)
*Bacillus licheniformis*	Water from oil well	Lipopeptide biosurfactant	Lignite	Solubilization occurred with a 53,000 Da component of lignite	Biosurfactants have the potential to facilitate coal refining	(Polman et al. 1994)
*Penicillium decumbens* P6	Coal mine soil	Biosurfactant containing cell-free filtrates	Lignite	The activity of lignite-attacking enzymes was increased by biosurfactants	Biosurfactants were 10 times more effective than the artificial products	(Yuan et al. 2006)
*Penicillium ortum* MJ51	Lignite from coal mine	Extracellular surfactants	Lignite	Alkaline substances and surfactants were the primary contributors to lignite depolymerization	*P. ortum* MJ51 exhibits promising potential for production of lignite-based value-added products	(Li et al. 2023)
*Pseudomonas fluorescens*	NS	Extracellular surfactants	Anthracite	Surfactants reduced surface tension and improved coal solubility	The mechanism by which cellular components degrade coal is established	(Fakoussa 1988)
*Pseudomonas otitidis* P4	Coal mining site	Glycolipid biosurfactant	NS	Biosurfactant production and emulsification contributed to the bioavailability of pyrene	*P. otitidis* P4 is potential for PAHs bioremediation and scale-up biosurfactant production	(Singh and Tiwary 2016)
Actinomycetes	Various soils	Different biosurfactants	Coal-vitamin medium	Coal was used as only nitrogen and carbon source for microbial growth	A method was proposed for selectively isolating biosurfactant-producing actinomycetes	(Ayoib et al. 2023)
*Bacillus aryabhattai* SPS1001	NS	Glycolipid biosurfactant	Waste coal tar	Nutrient medium with waste coal tar produced maximum yield of biosurfactant (0.43 g L^−1^)	Potential for biosurfactant-mediated bitumen recovery from oil sand	(Singh 2023)
*Bacillus* Lz-2	Oil-polluted water	Rhamnolipid biosurfactant	PAHs containing solution	Biosurfactant was applied to enhance PAH solubilization	An effective tool for removing petroleum and coal compounds from polluted sites	(Li et al. 2015)

*NS *not specified*, PAHs* polycyclic aromatic hydrocarbons

**Fig. 2:**
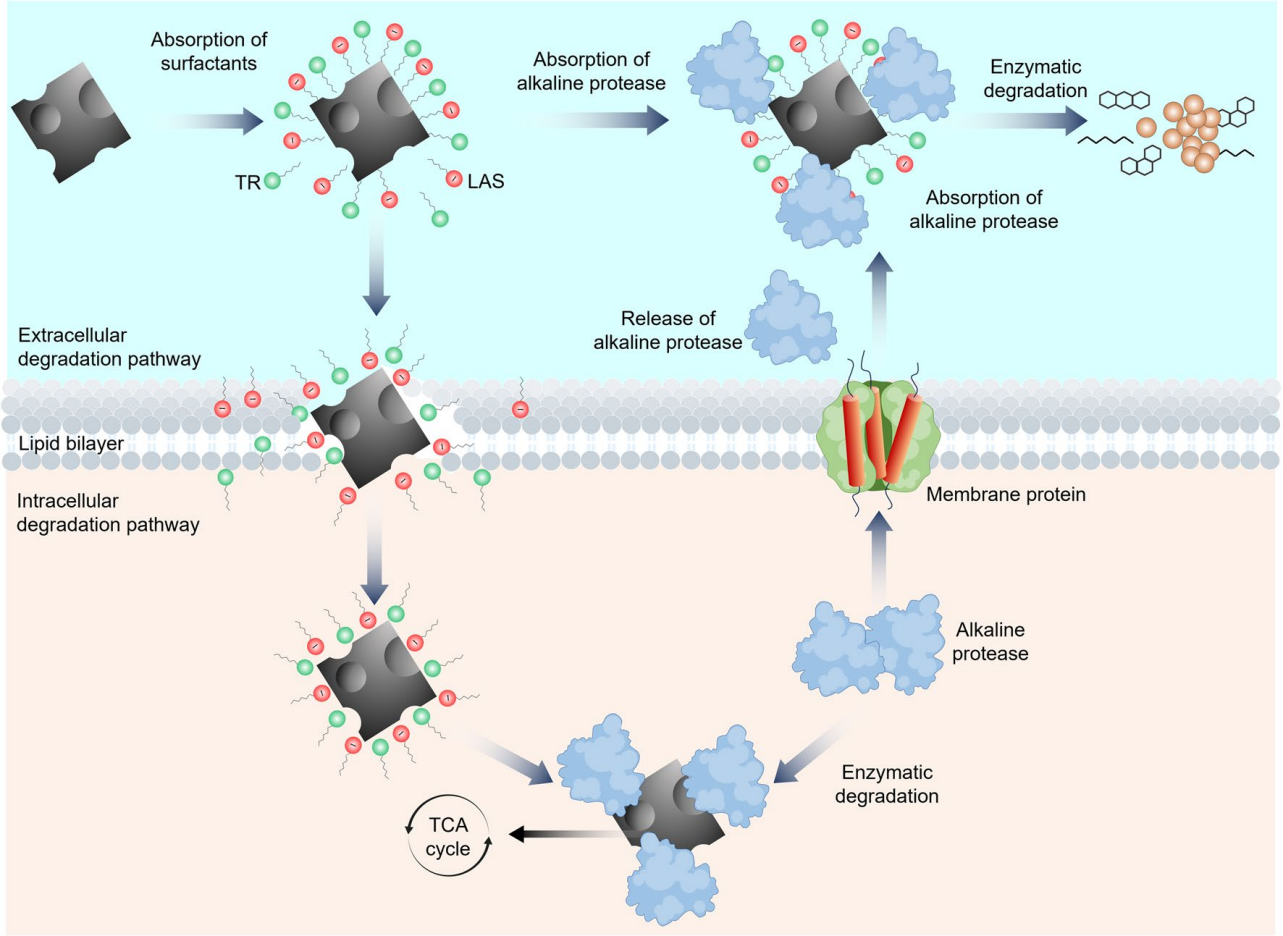
Possible mechanisms of coal biodegradation by a surfactant mixture (TR-LAS). Abbreviations: TR, octyl phenoxy poly ethoxy; LAS, sodium dodecyl benzene sulfonate; TCA cycle, tricarboxylic acid cycle

The surfactants used for coal biosolubilization are mostly anionic and nonionic. Nonionic surfactants may enhance microbial growth and exhibit superior stability; however, they are costly and require large dosages. Anionic surfactants readily adhere to the coal surface but they may exhibit poor solubility (Pardhi et al. 2022). The utilization of surfactant mixtures to improve the effectiveness of coal biodegradation shows great promise. Shi et al. achieved the highest coal biodegradation using the synergistic effect of the nonionic surfactant TR (octyl phenoxy poly ethoxy) mixed with anionic surfactant LAS (sodium dodecyl benzene sulfonate) (Shi, Liu, Wu, et al., 2023a). They described two possible mechanisms of coal biodegradation pathways via surfactant mixtures: intracellular and extracellular one. The graphical representation of the proposed mechanisms is given in Fig. 2.

Experimental results by Shi et al. and other research groups suggest that the mixed surfactant TR-LAS enhances the availability of –OH and –NH_2_ functional groups on the bacterial cell surface and at the same time improves the hydrophilicity of coal. This promotes the adsorption of bacteria and their secretions onto coal surfaces. One of the main indicators of coal biodegradation is the secretable microbial alkaline protease, whose activity is strongly correlated with the rate of coal transformation. According to the scheme illustrated in Fig. 2, within the extracellular degradation route, the hydrophilic portion of the TR-LAS mixture is attached to the oxidized coal, whereas the hydrophobic portion is attached to alkaline protease. Consequently, alkaline protease facilitates the breakdown of oxidized coal into smaller molecules such as long-chain alkanes and aromatic compounds. In the intracellular degradation route, coal samples coated with TR-LAS first penetrate the phospholipid bilayer. Reverse micelles formed in the phospholipid bilayer allow coal samples to enter the cytoplasm. Inside the cytoplasm, alkaline protease helps degrade coal particles into smaller molecular that can then enter the tricarboxylic acid (TCA) cycle.

Many studies published so far suggest that the direct application of surfactants is an effective method for accelerating the rate and reducing the duration of coal biodegradation (Table 2). Analysis of the data in Table 2 indicates that this approach features cost-effectiveness and simplicity of operation.

**Table 2:** Published studies on direct surfactant applications in coal biosolubilization

Surfactant	Producing microorganism	Coal type	Reported effects	Mechanism of action	Ref
Rhamnolipid	*Pseudomonas japonica*	Inner Mongolia coal	The mixture of rhamnolipid and esterase significantly promoted coal biodegradation	Rhamnolipid increases coal surface, making it more amenable to esterase-coal interaction	(Shi et al. 2023b)
SDS	Extracellular proteins of fungus AH	Fushun mine coal	Lack of surfactants resulted in a reduced amount of enzymes being attached to the coal surface	Surfactants changed the coal surface’s charges and hydrophilic property to allow more enzymes to solubilize	(Yin et al. 2011)
Rhamnolipid	*Pseudomonas* sp. Ph6	PAHs containing solution	Rhamanolipid altered Ph6 cell envelope microstructures and functional groups, improving cell membrane potential	Rhamnolipid enhances the cell-surface zeta potential and hydrophobicity, thus consequently degrade PAHs	(Ma et al. 2018)
Anionic and nonionic surfactants	*Nocardia mangyaensis*	Wucaiwan coal	Significant cell growth was observed in the presence of mixed surfactants	Surfactants secreted more alkaline proteases and rendered the coal more compatible with *N. mangyaensis*	(Shi, Liu, Wu, et al., 2023a)
SDS, LAS, Tween 80, and Triton X-100	*Ochrobactrum cytisi*, *Novospingobium naphthalenivorans*, *Alcaligenes faecalis*, and *P. fluorescens*	Shenmu lignite	*P. fluorescens* achieved the highest coal solubilization rate of 61.9% via Triton X-100	Adding Triton X-100 could reduce the contact angle between oxidized coal and surfactant solutions	(Kang et al. 2021)
Tween 80, Triton X-100, and Brij30	*Sphingomonas* sp. GY2B	PAHs	Tween 80 enhanced the biodegradation of PAH	Tween 80 has a positive impact by serving as an extra carbon source for GY2B	(Liu et al. 2016)
SDS	*Hypocrea lixii* HN-1	Bituminous coal	Pretreatment by SDS increased coal biosolubilization	Small molecule functional groups were introduced into the coal following pretreatment	(He et al. 2022)
Tween-80	*Phanaerochaete* *chrysosporium* (J1) and *Trametes versicolor* (J2)	Weathered coal	Coal biosolubilization was enhanced by 15% (J1) and 12% (J2) along with Tween-80	Surfactant may increase the production and consumption of humic acids	(Tong et al. 2024)
Triton X-100, SDS and DTAB	P. aeruginosa	Low-rank coal	Coal biodegradation rates reached 78.63% (Triton X-100), 55.93% (SDS), and 20.93% (DTAB)	Triton X-100 increased coal degradation rate by increasing cell membrane permeability	(Wu et al. 2022)
Rhamnolipid, Triton X-100, LAS and DTAB	*Bacillus licheniformis*	Tar-rich coal	Triton X-100 demonstrated the most significant enhancement in coal hydrophilicity	The degradation of aromatic and aliphatic structures of coal was accelerated by Triton X-100	(Shen et al. 2023)

*SDS* sodium dodecyl sulfate, *PAHs* polycyclic aromatic hydrocarbons

## Surfactants in biogenic coal-to-methane conversion

Coalbed methane (CBM) is gaining economic and scientific attention due to the continuously escalating demand for energy and the consequent fast depletion of conventional energy resources. The majority of methane contained within CBM deposits is generated microbially. However, the bioconversion of coal to methane is a relatively slow natural process owing to the complex chemical nature of coal (Park and Liang 2016; Sharma et al. 2018). To increase process efficiency, various coal pretreatment techniques are employed—many of them use oxidants, surfactants, chelating agents, acids, and alkalis. Surfactants, by lowering the surface tension and hydrophilicity of coal, may improve coal dissolution and accelerate its biodegradation. Additionally, higher coal biodegradation rates may result from the surfactant’s ability to alter the reaction sites of certain enzymes. Prior to methane production from coal, extracellular biosurfactants secreted by microorganisms interact with coal components, thereby increasing their solubility in water (Davis and Gerlach 2018; Faiz and Hendry 2006).

Existing research indicates various biosurfactant-producing microorganisms in methane-bearing coal environments, implying the key role of biosurfactant-production in improving coal bioavailability (Table 3). Schweitzer et al. examined environmentally relevant metagenomes from coal seams in the Powder River Basin with the aim of identifying genes and functional clusters involved in coal degradation. They showed that the biosurfactant genes associated with surfactants such as surfactin and lichysein were especially abundant in metabolically active microbial populations, suggesting a significant role of these biosurfactants in coal biogasification (Schweitzer et al. 2022). According to the study by Singh and Tripathi, coal addition to a medium containing bacteria isolated from coal-formation water led to a significant increase in biosurfactant production (Singh and Tripathi 2013). Zhang et al. (2018), used a specially designed 3-L fermenter to measure coal biogasification. Their findings revealed that the fermentation broth was rich in aromatic compounds and fatty acids, among other chemical compounds. Furthermore, the broth contained substances exhibiting biosurfactant properties that lowered surface tension to 54.5 ± 2.2 mN/m, which is lower than that of pure water (~ 72 mN/m).

**Table 3:** Published reports demonstrating the correlation of microbial biosurfactant production (or chemical surfactant addition) with coal solubilization and subsequent CBM production

Producing microorganism	Surfactant	Coal type	Reported effects	Prospects/outlook	Ref
*Pseudomonas stutzeri* isolated from coalbed formation water	Rhamnolipid	Lignite	Biosurfactant exhibited emulsifying ability and stability across a broad pH, temperature, and salinity	*P. stutzeri* potential to facilitate in situ biotransformation of coal into methane within the coalbed	(Singh and Tripathi 2013)
*Actinobacteria* from coal core samples	Biosurfactant and Tween 80	Core coal	The bioassay found that the coal was more bioavailable due to the biosurfactant, and adding nutrients promoted its in situ production	Contributes novel insights into the in situ bacterial communities linked to coal and potential pathways implicated in coal degradation	(Barnhart et al. 2016)
Viable microbial consortia from coal seam formation water	Zonyl FSN, Triton X-100, and Brij 35	Sub-bituminous coal	Zonyl FSN addition improved initial methane production by 240% and final methane yield by 180%	An opportunity to augment coalbed methane reserves through the utilization of in situ stimulation	(Papendick et al. 2011)
Methanogenic community	Surfactin	Lignite and bituminous coal	Surfactin yielded from bituminous coal (0.3901 ± 0.0027 SD) and contributed to enhancing its bioavailability	Enables efficient biomethane production via suitable metabolic strategies	(B. Wang et al. 2021a, b)
In situ coal-associated biofilms	Lichysein, rhamnolipids	Coal seam samples	Various biosurfactant genes were most present in samples	In-depth knowledge of subsurface coal degradation strategy	(Schweitzer et al. 2022)
NS	Fluorocarbon surfactant PMP	Coal core samples	Water-to-gas wetness conversion of the coal increased gas-flooding water recovery by 20.31%wt	Gas wettability alteration approach can enhance coal-bed methane production	(Jia et al. 2019)

*NS* not specified, *PMP* perfluorooctyl methacrylate monomer–containing polymethacrylate

## Surfactants in coal biobeneficiation

Biosurfactants produced by various microorganisms possess the capability to alter the surface characteristics of minerals because of their amphiphilic organic structure. This property plays a crucial role in mineral beneficiation. Likewise, the combination of synthetic surfactants and microorganisms can provide efficient technological solutions for mineral biobeneficiation (Abhyarthana and Rayasam 2019).

In their recent review, Asgari et al. (2024) classified microorganisms commonly utilized in mineral processing into two main process categories: “biomining” and “biobeneficiation,” where “Biomining” comprised two sub-groups: *bioleaching* and *biooxidation*. Although these two terms are frequently used synonymously, bioleaching refers to the dissolution of insoluble metals and the transfer of the target metal into a solution, while biooxidation primarily refers to the microbial decomposition of minerals without solubilizing the metal. In contrast, biobeneficiation encompasses the processes of *bioflotation* and *bioflocculation*. Here, microorganisms, functioning as reagents, collectors, or modifiers, facilitate separation selectivity in the context of these two processes. The majority of research examining the relationship between surfactant-targeted microbial activity and coal processing has focused on bioflotation (see the “Surfactants in coal bioflotation” section), whereas bioflocculation has received comparatively less attention so far.

### Surfactants in coal bioleaching

Bioleaching, a process employed to dissolve/extract valuable metals from mineral resources and eliminate impurities such as sulfur from coal, is more specifically termed *biodesulfurization* (see the “Surfactants in coal biodesulfurization” section). Various bacterial strains, including *Acidithiobacillus ferrooxidans*,* A. thiooxidans*, and *Bacillus mucilaginosus*, have shown selective vanadium-leaching effects with respect to vanadium-bearing coal (Tupikina et al. 2013). There are techniques developed to speed up microbial leaching and increase the effectiveness of vanadium recovery, such as mineral roasting and fortification agent addition. Among them, surfactants have proven effective in accelerating the ore leaching rate, improving the ore surface’s wettability and increasing the ore pile permeability (Fang et al. 2014). In a study by Dong et al., SDS was used as a leaching enhancer for *B. mucilaginosus* in leaching tests on vanadium-containing coal. Their findings indicated that the appropriate dosage of surfactant (0.1 g/L) improved the properties of the mineral surface and that SDS helped in creating an optimal acidic environment, thus ensuring a smooth leaching process (Dong et al. 2023).

According to another study of Dong et al., the highest vanadium extraction rate of 30.1% was reached at an SDS concentration of 0.05 g/L in the presence of *B. mucilaginosus* (Dong et al. 2019). When higher surfactant concentrations were applied, the metal dissolution efficiency decreased, possibly due to inhibition of bacterial growth. Similarly, the application of Tween-20 surfactant first increased the leaching rate, but subsequently decreased it depending on the Tween-20 dosage (Dong et al. 2019).

### Surfactants in coal bioflotation

The utilization of low-rank coals (LRC), especially oxidized ones, poses major environmental challenges due to their high ash content and low calorific value. Thus, improving the effectiveness of LRC combustion and reducing pollution are of great significance. Flotation, a very powerful particle separation technique, is very suitable to eliminate impurities and improve the quality of coal. Regrettably, (oxidized) LRC expose numerous hydrophilic functional groups (–COOH and –OH) on their surface, which hinder the adsorption of oily collectors and consequently diminish the flotation efficiency by forming stable hydration films (Xue et al. 2023). To increase the hydrophobicity of the coal surface prior to LRC flotation, a variety of techniques, such as chemical pretreatment, dry pulverizing, heat treatment, microwaving, and ultrasonic treatment, is implemented (Asgari et al. 2024). Nevertheless, the use of these techniques is constrained by many factors, such as increased operating costs, excessive use of aggressive chemicals, adverse environmental effects, and limited applicability (Mishra et al. 2023). *Bioflotation* has garnered growing interest as an addition/alternative to conventional techniques because of its notable selectivity, eco-friendliness, and cost-effectiveness. Microbial cells and their metabolites can serve as efficient frothers (forming the micro-bubbles), collectors, or depressants in bioflotation processes. Bioflotation methods can be categorized into *direct* (where microorganisms are directly applied to treat coal) and *indirect* (where microbial metabolites are used instead of microorganisms) variants (Asgari et al. 2024).

Biosurfactants, with their unique properties, confer significant advantages in the bioflotation process. Their amphiphilic structure allows them to enhance the wettability and reactivity of hydrophobic substances and to adjust the surface characteristics of bacterial cells. Due to their surface activity, biosurfactants are excellent foaming, dispersing, and emulsifying agents. Primarily utilized as frothers in the bioflotation process, biosurfactants create froth with the desired stability (Khoshdast et al. 2023). Enhanced surface activity is correlated not only with higher froth stability, but also with better *frothability* (which can be measured as froth height) (Didyk and Sadowski 2012; El-Midany and Abdel-Khalek 2014). In some cases, as reported by Dhar et al. and Augustyn et al., biosurfactants were used in the flotation process as depressants and collectors (Dhar et al. 2021; (Gholami and Khoshdast n.d.).

**Fig. 3:**
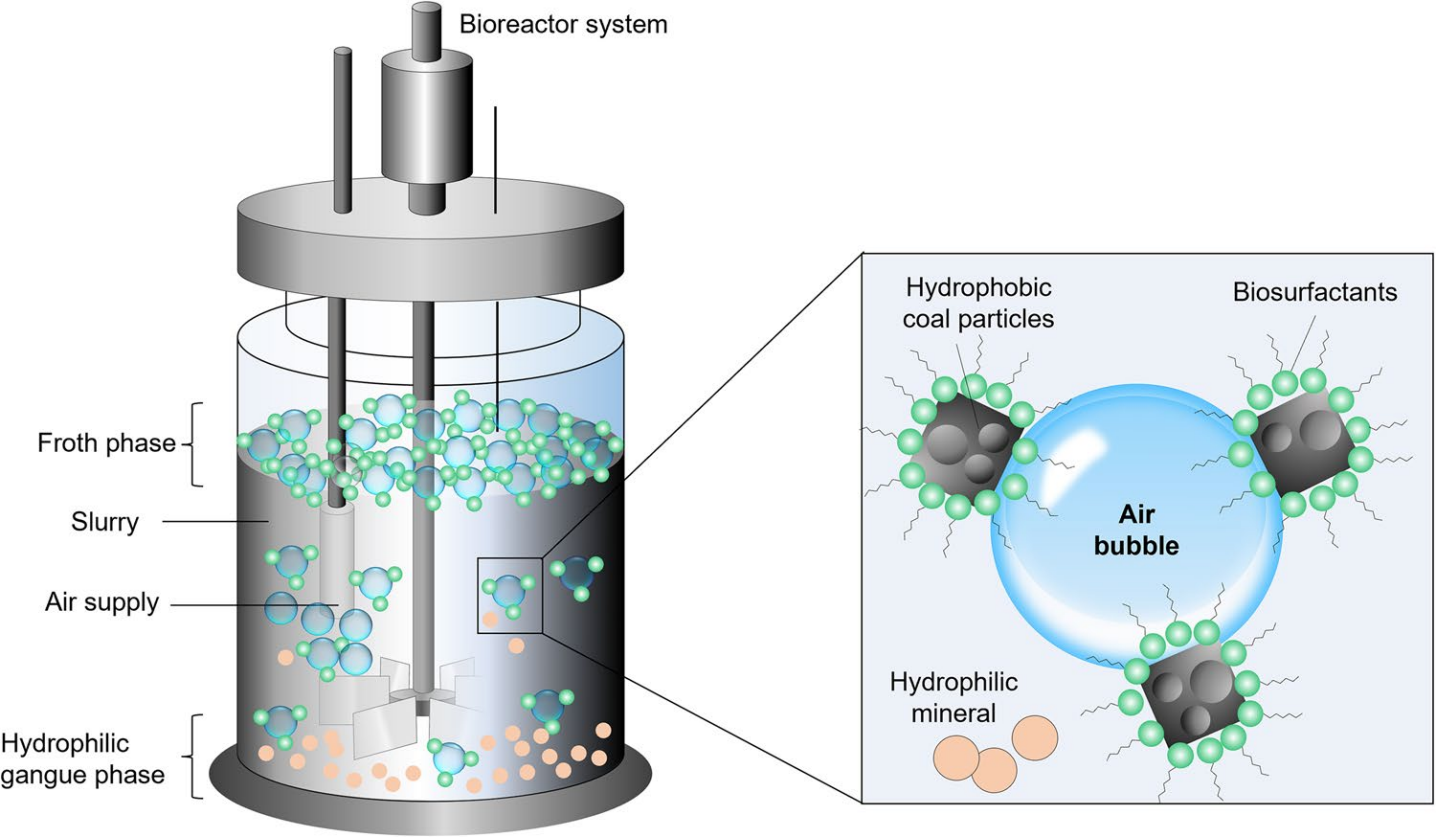
Schematics of a coal bioflotation reactor (left) and coal particle–rhamnolipids adsorption (right). The nonpolar head of the surfactant molecule interacts with the air/water interface, whereas the polar end forms hydrogen bonds with water. This process uses changes in water surface tension, those magnitude serves as an indicator of the surface activity of surfactants

Figure 3 illustrates the bioflotational separation of hydrophobic coal particles from hydrophilic gangue minerals using microbial rhamnolipids as frothers. Rhamnolipid molecules adsorb at the air–water interface due to their folded structure, with two hydrophobic chains oriented toward the air bubbles. In turn, the interaction between the polar hydrophilic heads can generate a film that is even more densely packed than that of ethers and alcohols.

There are many studies demonstrating that bioflotation, in which biosurfactants were combined with conventional moist techniques, can successfully and selectively purify coal materials (Table 4).

**Table 4:** Examples of coal purification by flotation using biosurfactants derived from microorganisms

Technology	Biosurfactant	Coal type	Study type	Results reported	Ref
Froth flotation	Surfactin from *Bacillus subtilis*	Sub-bituminous, South Africa	The surface interaction between surfactant and coal	Hydrophobicity of coal was increased by surfactant at pH 3 and 15 mg/L	(Augustyn et al. 2021)
Mineral flotation	Rhamnolipid from *Pseudomonas aeruginosa* MA01	Coal, Iran	Surfactant application as a frothing agent in coal and hydrophilic mineral flotation	Surfactant decreased the ash content and coal yield of concentrates	(Khoshdast et al. 2011)
Cadmium removal	Rhamnolipid from *Pseudomonas aeruginosa* MA01	Coal waste, Iran	Using depressing effect of surfactant to activate coal waste for removal of heavy metals	Absorbent to cadmium ratio of approx. 125 and 10 h contact removed over 99% cadmium at pH 9	(Boveiri Shami et al. 2019)
Ash removal	Rhamnolipid from *Pseudomonas aeruginosa*	Semi bituminous coal, Iran	Impact of surfactant on the ash removal efficiency by froth flotation	Surfactant showed low selectivity and high frothability in terms of yield/ash correlation	(Khoshdast and Shojaei 2012)
Froth flotation	Rhamnolipid from* Pseudomonas aeruginosa*	Coal, Iran	Surfactant production and its frothing characterization	The surfactant had high frothability in terms of lowering surface tension, froth height/stability	(Fazaelipoor et al. 2010)
Bioflotation and artificial neural network	Rhamnolipid from *Pseudomonas aeruginosa* MA01	Coal, Iran	Importance of how surfactant impacts the efficiency of coal flotation by statistical analysis	Surfactant had a depressing effect on coal flotation	(Gholami and Khoshdast n.d.)
Collector and frother	Biosurfactant-like substances from *Bacillus*	Coking coal, China	Influence of native microorganisms on coal flotation	Substances enhanced froth stability by reducing water entrainment	(X. Wang et al. 2023a, b)
Heavy metal removal	Biosurfactants from *Candida lipolytica* UCP0988 and *C. sphaerica* UCP0995	AMD near coal mine, Brazil	Applying surfactants as alternative collectors in flotation systems	The surfactants achieved a removal effectiveness rate of 90% after 15 min	(Albuquerque et al. 2012)
Bio-liquefaction and bioflotation	Biosurfactants from *Hypocrea lixii* AH	Lignite, China	Exploring AH as a bioflotation agent in lignite utilization	Surfactants reduced the concentration or enhances the flotation of lignite impurities	(He et al. 2023)
Benefi-ciation	Biosurfactants from microalgae	Lignite, Indonesia	Lignite conversion with biosurfactant	The calorific value of lignite was improved	(Mahreni and Puspitasari 2020)

*AMD* acid mine drainage

## Surfactants in coal biodesulfurization

Despite the economic significance of coal, its combustion is accompanied by detrimental environmental issues, including sulfur oxides (SO_X_) emission, acid rains, and toxic airborne particle formation. Air pollution by SO_X_ (especially by SO_2_) discharge exerts harmful impacts on living organisms and their environment. Obviously, reducing the sulfur content in coal prior to combustion can drastically contribute to resolving SO_X_ emissions. Biodesulfurization techniques employing microorganisms have aroused immense interest as a novel sustainable and environmentally friendly approach for removing sulfur (specifically organic and pyritic sulfur) from coal (Çelik et al. 2019).

**Fig. 4:**
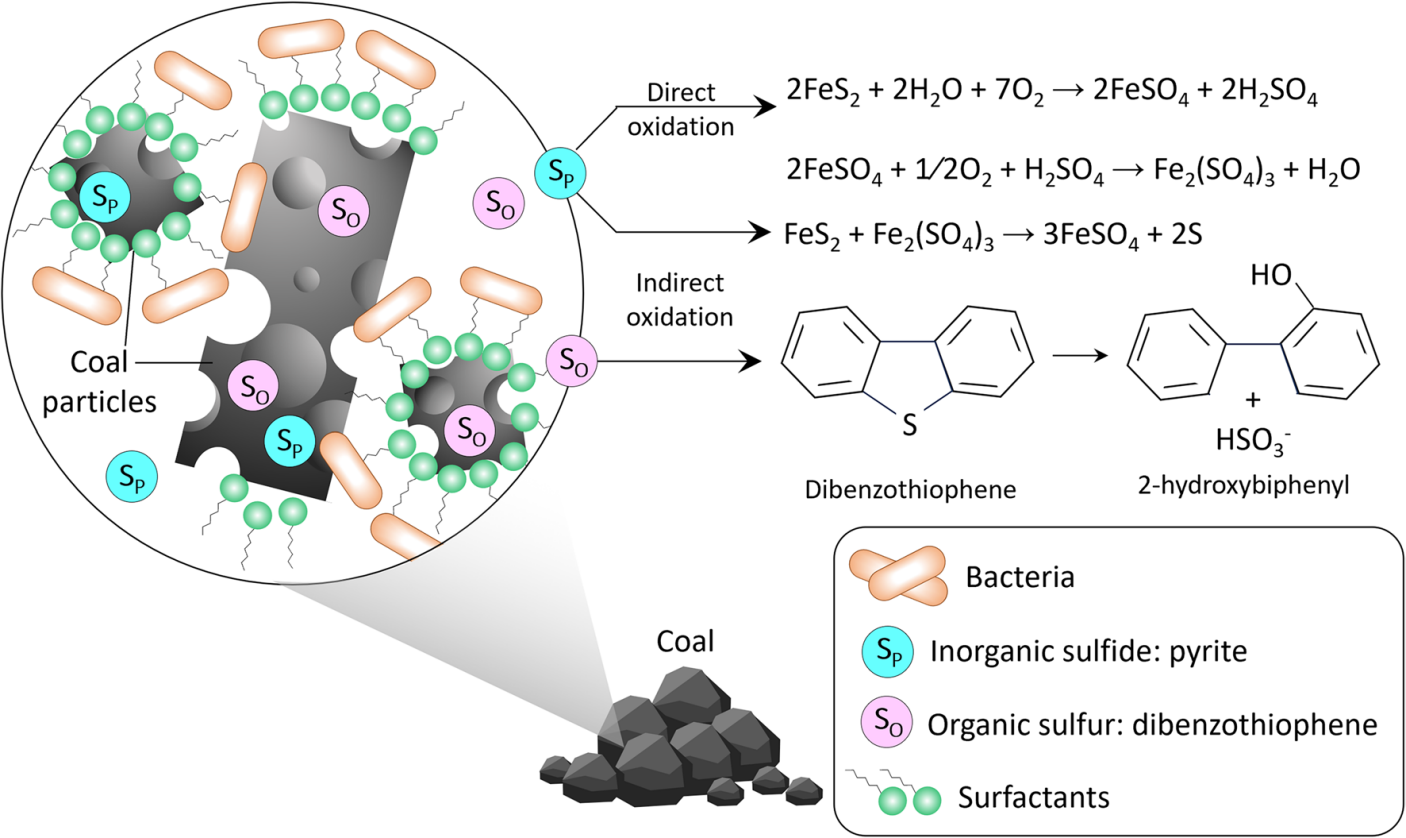
Possible mechanism by which surfactants influence the coal biodesulfurization efficiency of bacteria

Recent research on biodesulfurization activity enhancement has shed light on the potential of surfactants to improve sulfur removal efficiency (Table 5). Surfactants, being amphiphilic compounds, help improve the mass transfer of the system, thereby accelerating the desulfurization process. By decreasing surface and interfacial tensions, surfactants can alter the solubility of hydrophobic compounds and enhance their transport through bacterial membranes, promoting the desulfurization rate (Mishra et al, 2018b). The presence of surfactants results in surface modifications leading to a more favorable interaction between the microbial cell and coal, which is beneficial for biodesulfurization performance (Mishra et al., 2018c). A schematic representation of typical coal biodesulfurization reactions augmented by surfactants is shown in Fig. 4. The desulfurizing microorganisms may utilize a surfactant and simultaneously attack the coal matrix to fulfill their sulfur needs. In the presence of surfactants, two types of sulfur in coal become more bioavailable: inorganic sulfides S_p_ (e.g., ferrous sulfide–pyrite) and organic sulfur compounds—S_o_.

**Table 5:** Surfactants in coal biodesulfurization: recent case study reports

Microorganism	Surfactant	Sulfur compound	Results reported	Mechanism of action	Ref
*Rhodococcus* *erythropolis* DSM 44308	Span 80	Lignite (S_o_ = 2.59%)	44.6% of desulfurization using 2% v/v Span 80	*R.erythropolis* utilized the extra surfactant and attacked the coal matrix to fulfill its sulfur needs	(Mishra et al. 2018c)
*Rhodococcus erythropolis*	Tween 80	DBT	The highest activity was reached at 1 µmol g^−1^ min^−1^ when Tween 80 above CMC	Tween 80 may improve desulfurization activity by decreasing the production of inhibitory compounds	(Feng et al. 2006)
*Corynebacterium sp.* ZD-1	Brij-35, Tween 80, Triton-100X, and β-cyclodextrin	DBT	2-Hydroxybiphenyl formation was increased by 50% with Tween-80 compared to control	Tween 80 may enhance the mass transfer of DBT between organic and aqueous phases	(Wang et al. 2006)
*Acidithiobacillus* *caldus* and *Acidithiobacillus thiooxidans*	SDS, DTAB, and Tween 20	Coal (S_tot_ = 4.55%)	When 1100 mg/L of Tween 20 was added, the overall desulfurization rate reached 29.7%	Tween 20 enhanced bacterial adsorption, leading to increased extracellular polymeric substance release	(Zhang et al. 2017)
*Thiobacillus ferrooxidans*, *Escherichia coli*, and *Pseudomonas putida*	Tween 80	Coal (S_tot_ = 5.05%)	*P. putida* removed 58.23% of S_tot_ with 0.1% Tween 80	Tween 80 facilitated the mitigation of interfacial tension, thereby enhancing the affinity between coal and microorganisms	(Xu et al. 2020)
*Leptospirillum ferriphilum*	Span 80	Lignite (S_o_ = 7.63%)	Span 80 (0.05% v/v) removed nearly 61% of S_tot_	Span 80 may enhance coal bioavailability for biodegradation	(Mishra et al. 2018b)
*Leptospirillum ferriphilum*	Span 80	Lignite (S_o_ = 7.63%)	S removal was lower with Span 80 by 9% under continuous fermentation	Fe^2+^ may react with Span 80, resulting in the inhibition/slowing down of the Fe^2+^ oxidation	(Mishra et al. 2018a, b, c)
Bacterial consortium IQMJ-5	Tween-20, Tween-80, SDS, and EDTA	DBT	Tween 80 showed high desulfurization rate about 66%	Tween 80 had the least inhibitory property on bacterial metabolism and growth	(Khan et al. 2022)

*DBT* dibenzothiophene, *CMC* critical micelle concentration

Fernando Bautista observed a significant enhancement in the biodesulfurization capabilities of microorganisms upon the addition of surfactants above the critical micelle concentration (CMC). They attributed this effect primarily to the increased growth of bacteria in the presence of surfactants, which serve as an additional carbon source for bacteria (Fernando Bautista et al. 2009). Furthermore, surfactants can facilitate the transfer of chemicals between the aqueous and organic phases, thereby increasing coal accessibility for microbial attack (Wang et al. 2006).

Biosurfactants are proposed to play a “strategic role” in reducing the sulfur content in coal. Handayani et al. investigated the biodesulfurization of organic, pyritic sulfur, sulfate, and total sulfur in Tondongkura coal through a multi-stage bioprocess treatment employing *Pseudoclavibacter *sp. strain SKC/XLW-1, known for its biosurfactant production capabilities (Handayani et al. 2017). Their findings suggest that SKC/XLW-1 and its metabolic products are key players in sulfur removal from coal due to their ability to secrete biosurfactants and oxidize sulfur simultaneously.

## Surfactant-aided microbial coal dust suppression

Coal mining, refining, and transportation give rise to elevated levels of particulate matter (dust) in the atmosphere, posing significant hazards such as pollution, explosion, and spontaneous combustion. Exposure of residents and workers to hazardous coal dust concentrations significantly increases their health risks (Liu and Liu 2020).

Coal dust suppression based on microbially induced calcium carbonate precipitation is a relatively new technique that has gained popularity owing to its low cost, minimal environmental impact, and high particle aggregation efficiency. The fundamental principle behind this technology, thoroughly described by Seifan et al. in their recent review, is related to urea hydrolysis combined with biosurfactant action (Seifan and Berenjian 2019).

The inherent hydrophobicity of coal dust particles arises from numerous non-polar groups (aromatic and aliphatic) on their surface. Surfactants, being amphiphilic compounds, can decrease the hydrophobicity of coal’s surface and consequently significantly mitigate the hazards associated with coal dust (K. Wang et al. 2019a, b, c; Wang et al. 2020). As the particle size of coal dust decreases, its wettability diminishes, and its microstructure becomes increasingly complex. The insufficient wettability of coal dust can be successfully remedied by applying a surfactant (Li et al. 2013). In the absence of surfactants, the microbial dust suppressant forms a thin layer of CaCO_3_ particles, which subsequently compromises the ability of crusts to withstand external forces and eliminates the dust suppression effect. Surfactants contribute to the process in many ways: (a) they improve wetting performance by adsorbing onto the dust particle’s surface; (b) they aid in the dispersion of particles in the suppressant solution, thus facilitating better coverage of the coal dust surface; and (c) they help stabilize particle coatings by promoting the formation of more robust bonds between the aggregating agent and the dust particles. According to two recent studies, the suppression properties of microbial dust suppressants containing surfactants are between 29.61 and 31.98 times greater than those without surfactants (Y. Zhao et al. 2023a, b; Zhu et al. 2021).

Currently, there is a methodological trend toward replacing synthetic surfactants with bio-analogs, as the latter display an environmentally “benign” nature, rapid biodegradability, and minimal toxicity (Eras-Muñoz et al. 2022). Considerable research has been conducted in recent years on the impact of various surfactants and their combinations on the efficacy of urease-producing microbial dust suppressants (Table 6).

**Table 6:** Recent studies devoted to (bio)surfactant-assisted in microbial coal dust suppression

Microorganism	Surfactant/biosurfactant	Coal type	Performance analysis	Results reported	Rate effects	Ref
*Pseudomonas* PLD01	Rhamnolipid from PLD01	Coal from Suncun Coal Mine	Emulsification index, PLD01 biosurfactant production, biosurfactant characterization	The biosurfactant extracted by PLD01 exhibited superior wettability to coal in comparison to SDS	PLD01 decreased the surface tension of water from 72.8 to 40.6 mN/m	(Liu et al. 2022)
*Bacillus* X4	CAB	Bituminous coal from Shandong Longyun Coal Industry Co. Ltd	Adsorption experiment, dust suppression, wind erosion experiments	The surface tension was most drastically decreased by the combined effect of bacteria and surfactants	The adsorption capacity of X4 was the highest for 40–80 mesh coal (40.71 mg/g)	(Zhang et al. 2022)
*Bacillus cereus* CS1 and *Sporosarcina pasteurii* ATCC11859	SDBS, CTAB, CAB, and APG	Coal from Shandong Longyun Coal Industry Co., Ltd	Surfactant screening, dust-suppression property of suppressant	CAB-microbial dust suppressant exhibited enhanced resistance to wind erosion and evaporation	The combined efficiency was 2.40–2.71 times than surfactant alone	(Zhu et al. 2021)
*Bacillus* X4 and* Pseudomonas* PLD01	Rhamnolipids from PLD01	Coal from Suncun Coal Mine	Co-culturing urea-hydrolysis X4 and biosurfactant PLD01	X4 provided fatty acids to produce biosurfactants, while PLD01 provided an alkaline environment for mineralization	P_14_X inoculation showed significant mineralization and emulsification	(Cheng et al. 2024)
Microbial community form a waste-activated sludge	SDBS, APG, and CAB	Coal dust	Wetting test, biomineralization, coal dust consolidation	SDBS and APG may decrease the pathogenic risk of microbial dust suppressants	The rain resistance/hardness indicated that 0.2% SBDS had the best effect	(Y. Zhao et al. 2023a, b)
NS	Bio-based rhamnolipid, APG, SDBS, CAB, SLS, OP-10	Coal from Wangpo mining area	Surfactant selection, surfactant characterization, and performance evaluation	An environmentally friendly and non-polluting rhamnolipid-based dust suppressant mixture was developed	The surface tension of mixture reduced to 23.95 mN/m, the contact angle to coal dust was 25°	(Niu et al. 2023)
*Bacillus* X4	CAB	Bituminous coal from Shandong Longyun Coal Industry Co. Ltd	Isotherm adsorption, adsorption kinetics, dust suppression, wind-rain erosion	Due to the CAB’s wetting effect, the contact area between the microbial suppressant and the dust increased	Higher dust content led to improved adsorption and consolidation effects	(Y.-Y. Zhao et al. 2023a, b)
NS	Rhamnolipid, lactan sophorolipid, surfactin,	Anthracite coal from Baoshan Yuan Coal Mine	Biological compound dust suppressant preparation, sedimentation, wettability	The ideal proportions for the dust suppressant were 12.9% rhamnolipid, 4.4% lactone sophorolipid, and 2.9% surfactin	28.515 mN/m was the minimum surface tension of the biodegradable dust suppressant	(K. Wang et al. 2021a, b)

*NS* not specified, *CAB* cocamidopropyl betaine, *SDBS* sodium dodecylbenzene sulfonate, *APG* alkyl polyglycoside, *SLS* sodium lauryl sulfate, *OP-10* alkylphenol polyoxyethylene ether

**Fig. 5:**
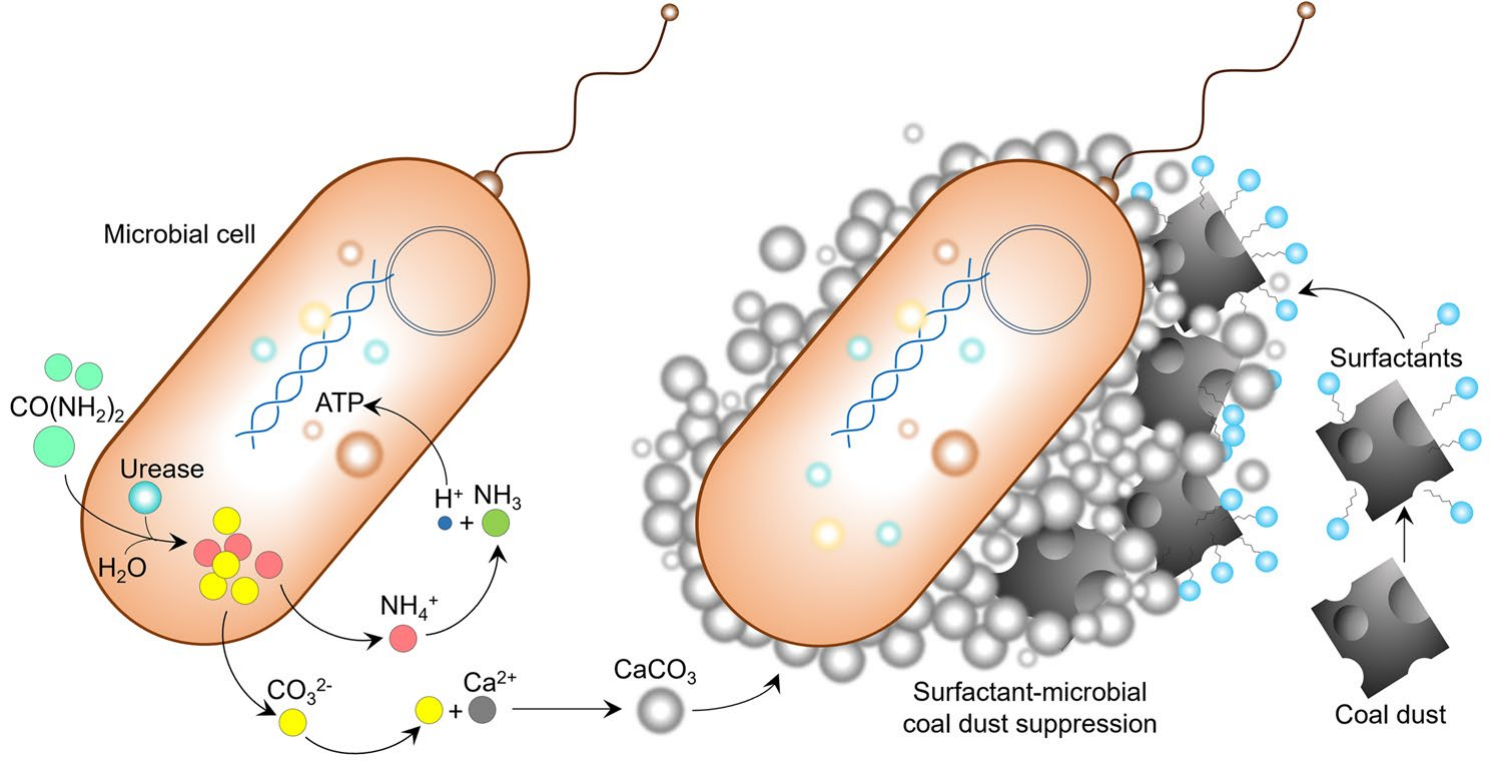
Surfactant-aided microbially induced coal dust precipitation mechanism. Abbreviations: ATP, adenosine triphosphate; CO(NH_2_)_2_, urea; CaCO_3_, calcium carbonate; CO_3_^−2^, carbonate anion; NH_4_^+^, ammonium cation; NH_3_, ammonia

The process of surfactant-aided coal dust suppression via urea hydrolysis operates as shown in Fig. 5. In this process, urea (CO(NH_2_)_2_) undergoes hydrolysis facilitated by the urease enzymes produced by microorganisms, resulting in the formation of carbonate anion (CO_3_^−2^) and ammonium cation (NH_4_^+^), which co-exist in water in dynamic equilibrium with ammonia (NH_3_). The microbial cell wall, which carries a negative charge, attracts cations (Ca^2+^) from the surrounding solution to form hardly soluble calcium carbonate (CaCO_3_), which is then precipitated in the vicinity of the microbial cell wall. As mentioned above, surfactants/biosurfactants from dust suppressants increase the wettability of coal dust particles and reduce the surface tension of the solution. Finally, the surfactant-microbial dust suppressant solution consolidates the coal dust, leading to microbially induced mineralization.

Biomaterials containing surfactants exhibit a robust growth trend for coal dust control, mainly from the perspective of biodegradability. Various innovative approaches have been proposed and utilized to improve the dust-suppression effectiveness of microbial dust suppressants. In a recent study, Cheng et al. (2024) conducted microbiological co-culturing to synergize the emulsification performance of biosurfactant bacteria with the mineralization performance of urea hydrolysis bacteria. Their findings indicated a distinct collaborative effect between the two bacterial strains: the urea-hydrolyzing bacteria provided fatty acids to enhance rhamnolipid synthesis by the biosurfactant producer, while the biosurfactant bacteria created an alkaline environment, promoting mineralization and enhancing CaCO_3_ generation. In another study, Lu et al. developed a wetting agent with exceptional wetting properties by combining various synthetic surfactants with *Sapindus mukorssi* saponin extract (Lu et al. 2021). Similarly, Wang et al. effectively synthesized a microalgae oil–based coal dust suppressant, showing excellent performance in hard water and displaying remarkable environmental tolerance (H. Wang et al. 2023a, b).

## Knowledge gaps and research priorities

The research outlined above has shed light on some fundamental concepts and practical applications of coal-microbial-surfactant systems aimed at improved energy production and environmental protection. However, while considering this topic, some critical considerations must be kept in mind:

- Although the technology for surfactant-mediated coal degradation has made impressive progress, there are still serious obstacles and challenges, for example, slow degradation rates (i.e., long degradation duration), which noticeably impede large-scale industrial applications. Microbial surfactants, on the other hand, are biodegradable, have high activity, are nontoxic, and remain stable under extreme conditions. Consequently, their enormous potential in the context of commercial implementation should be considered.
- It is crucial to evaluate the economic viability of using biosurfactants compared with conventional synthetic surfactants. Although they are more environmentally favorable, they may also be more costly to manufacture and maintain. Consequently, it is imperative to conduct a cost–benefit analysis prior to determining the appropriate use of these surfactants in coal utilization. In general, the abovementioned studies illustrate the potential of novel biosurfactants; however, there is a dearth of information regarding their economic feasibility and operational effectiveness.
- Identifying the optimal surfactant type and concentration across different coal processing technologies is essential, considering the diverse nature and properties of surfactants. Furthermore, it is crucial to pay significant attention while using large concentrations of biosurfactants due to their wide range of biological activities.
- Microbial functional activity during coal processing may be diminished by the interaction of microbial cells with chemical surfactants. Particularly, the area and fluidity of microbial cell membranes may alter due to the insertion, replacement, or dissolution of phospholipids and lipopolysaccharides by surfactant molecules (Górna et al. 2011).
- Concerns related to contamination may arise from the introduction of surfactants into the environment. Therefore, potential (environmental) toxicity and degradation pathways must be thoroughly examined prior to selecting a surfactant as a coal-processing tool (Edwards et al. 2003). Again, one option to overcome this problem could be to employ biosurfactant-producing microorganisms and their derivatives.
- Surfactant-mediated coal processing technologies are mainly conducted in laboratory-scale settings. The feasibility and efficacy of these systems in full-scale outdoor or natural settings remain undefined. The significant limitations, including incapacity in large-scale operations, high production costs resulting from costly substrates, and patent rights, must be taken into account to fully disclose the enormous benefits of biosurfactants.
- The majority of research on surfactant-mediated coal utilization currently focuses on lignite, while other varieties of LRC, such as leonardite (weathered coal) and coal waste/residue receive little attention. Therefore, more efforts should be made to promote better characterization of process efficiency across various coal types.

## Conclusion

(Bio)surfactant-mediated coal–microbial systems have shown very promising versatility and impressive performance potential in the energy sector and environmental protection, even though they have not been exploited for large-scale industrial applications yet. The mechanisms behind the bioavailability of coal remain incompletely understood owing to its heterogeneous structure and the intricate interplay between coal and microbial cells. Integration of appropriate surfactant into the processes of coal biosolubilization, coal biobeneficiation, and coal dust suppression can optimize the interaction between coal particles and microbial cells, thereby enhancing process efficiency. As a result, developing and implementing highly selective surfactants may aid in addressing rate-limiting stages and improve coal utilization processes. As concluding remarks, incorporating cutting-edge technological advancements, such as genetic engineering, nanotechnology, computational modeling, and downstream processing, into interdisciplinary research would optimize biosurfactant production. In addition, further investigation is necessary to comprehend the interaction between cells, biosurfactants, and coal matrices, with the aim of enhancing our understanding of their mode of action in the context of clean coal technologies.

## Data Availability

The authors declare that the data supporting the findings of this study are available within the paper. Should any raw data files be needed in another format, they are available from the corresponding author upon reasonable request.

## Abbreviations

*AMD*: Acid mine drainage
*APG*: Alkyl polyglycoside
*CAB*: Cocamidopropyl betaine
*CBM*: Coalbed methane
*CMC*: Critical micelle concentration
*DBT*: Dibenzothiophene
*LAS*: Sodium dodecyl benzene sulfonate
*LRC*: Low rank coals
*OP-10*: Alkylphenol polyoxyethylene ether
*PAHs*: Polycyclic aromatic hydrocarbons
*PMP*: Perfluorooctyl methacrylate monomer-containing polymethacrylate
*SDBS*: Sodium dodecylbenzene sulfonate
*SDS*: Sodium dodecyl sulfate
*SLS*: Sodium lauryl sulfate
*TCA*: Tricarboxylic acid
